# An analytical cross-sectional study to describe and compare the mental health status of doctors and medical undergraduates in selected institutions in Colombo, Sri Lanka during COVID-19 pandemic

**DOI:** 10.1192/bjo.2021.777

**Published:** 2021-06-18

**Authors:** Chathurie Suraweera, Iresha Perera, Priyanka Rupasinghe, Janith Galhenage

**Affiliations:** 1 Professorial Psychiatry Unit, National Hospital of Sri Lanka; 2 Department of Paediatric Neurology, Lady Ridgeway Hospital for Children, Colombo

## Abstract

**Aims:**

The aim of this study was to describe and compare the mental health status of doctors and medical undergraduates in selected institutions during COVID-19 pandemic.

**Method:**

A cross-sectional analytical study was conducted among doctors working in major tertiary care hospitals two of which, risk is unpredictable and high, the other where all patients are positive for COVID-19 and among medical undergraduates. The doctors were selected using disproportionate stratified sampling and medical undergraduates using stratified cluster sampling. Data were gathered using a Google form containing socio-demographic details, perception on the pandemic and the General Health Questionnaire-12(GHQ-12).

**Result:**

There were 468 participants in the study and among them 243(51.9%) were doctors. Mean age of the doctors’ is 34.54(SD = 7.43) years and more than half (50.06%) were in post graduate training. Majority were worried about their health (65%) and their loved one's health (90.1%). Among doctors 220(90.5%) felt that they have moderate or higher risk of acquiring COVID-19 and 15.6% would not have worked due to the risk. According to GHQ-12, 182(74%) doctors were psychologically distressed (mean GHQ = 12.64, SD = 4.54) and it was significantly associated with age less than 35 years (p = 0.039) and worry about interruption of their daily routines(p = 0.010).

The mean age of 225 medical undergraduates was 25.20 (SD = 1.34) years and 176(78.2%) of the participants were psychologically distressed (mean GHQ = 14.32, SD = 6.67). Majority (59.11%) believed that they are at high risk of getting COVID-19. Their distress was significantly associated with the worry about the impact of COVID-19 related restrictions on their daily routines (p = 0.000).

Binomial logistic regression confirmed that doctors were distressed due to impact on their income whereas both doctors and medical undergraduates were distressed due to impact on daily routines.

**Conclusion:**

Nearly three quarter of both doctors and medical undergraduates were psychologically distressed during COVID-19 pandemic. The worry was due to contracting illness, financial issues and the COVID-19 regulations.

